# Pavlovian conditioning demonstrated with neuromorphic memristive devices

**DOI:** 10.1038/s41598-017-00849-7

**Published:** 2017-04-06

**Authors:** Zheng-Hua Tan, Xue-Bing Yin, Rui Yang, Shao-Bo Mi, Chun-Lin Jia, Xin Guo

**Affiliations:** 1 Laboratory of Solid State Ionics, School of Materials Science and Engineering, Huazhong University of Science and Technology, Wuhan, 430074 P.R. China; 2 State Key Laboratory for Mechanical Behavior of Materials, Xi’an Jiaotong University, Xi’an, 710049 P.R. China; 3 School of Electronic and Information Engineering, Xi’an Jiaotong University, Xi’an, 710049 P.R. China; 4 Peter Grünberg Institute and Ernst Ruska Center for Microscopy and Spectroscopy with Electrons, Forschungszentrum Jülich, D-52425 Jülich Germany

## Abstract

Pavlovian conditioning, a classical case of associative learning in a biological brain, is demonstrated using the Ni/Nb-SrTiO_3_/Ti memristive device with intrinsic forgetting properties in the framework of the asymmetric spike-timing-dependent plasticity of synapses. Three basic features of the Pavlovian conditioning, namely, acquisition, extinction and recovery, are implemented in detail. The effects of the temporal relation between conditioned and unconditioned stimuli as well as the time interval between individual training trials on the Pavlovian conditioning are investigated. The resulting change of the response strength, the number of training trials necessary for acquisition and the number of extinction trials are illustrated. This work clearly demonstrates the hardware implementation of the brain function of the associative learning.

## Introduction

Human brains outperform digital computers in many tasks, for example, recognition of objects, linguistic comprehension, abstract reasoning, due to the complex neural network consisting of 10^11^ neurons interacting with each other through 10^15^ synapses^1^. Electronic synapses are regarded as the physical building block for a hardware based artificial neural network with the function of brain-like computing^2^. Quite encouragingly, memristive devices^3–6^ have been reported to efficiently emulate several neuromorphic and cognitive properties, such as all-or-nothing spiking of an action potential in neuristors^7^, synaptic plasticity (e.g. spike-timing-dependent plasticity (STDP) and metaplasticity)^8–16^, pattern learning^17, 18^ and Pavlovian conditioning^19–27^.

Pavlovian conditioning, also known as classical conditioning^28^ or associative learning^29^, is an associative type of the implicit memory^30, 31^, which is unconscious or procedural memory. The Pavlovian conditioning helps a biological body to prepare for an expected or likely event. It was firstly described by Ivan Pavlov in 1927^32–34^; after a training process of feeding and ringing a bell, Pavlov’s dog started to salivate to the bell ringing. In the case of Pavlov’s dog, the food is an unconditioned stimulus (US) that produces an unconditioned response (UR), i.e., salivation, while initially a neutral stimulus (NS), i.e., bell ringing, does not cause a similar response. After the training process of repeatedly activating NS before US, an association is established between NS and US, therefore, NS produces a similar response as US. Such a process of establishing association is called as acquisition. In this case, NS is referred to as the conditioned stimulus (CS) and the corresponding response as the conditioned response (CR). The association between CS and US can disappear, such a process is denoted as extinction. The Pavlovian conditioning shows following features: (1) acquisition by training trials when CS and US are presented close to each other in time^35, 36^, (2) extinction with CS applied alone, and (3) recovery, namely acquisition again by training process after the last extinction, which usually happens faster than before^37, 38^. The Pavlovian conditioning is one of the best understood learning processes, where learning and memory result from the change of the function and structure of neurons and their interconnection synaptic strength^31, 39^.

The previous demonstrations of the Pavlovian conditioning with electronic devices are all based on synapses and neuroplasticity^19–27^, but not all the above features have been addressed. Ziegler *et al*.^20^ and Moon *et al*.^23^ presented a neuromorphic circuit and mimicked the acquisition and extinction of the Pavlovian conditioning, but the temporal relations of CS and US were not considered. Li *et al*.^26^ demonstrated the Pavlovian conditioning with temporal contiguity, but the training process was quite simple without considering the learning frequency and the recovery features. Hu *et al*.^27^ focused on the demonstration of the associative memory on the basis of a memristive Hopfield network and the retrieval of pre-stored patterns, but the above features were not considered. These works are based on completely non-volatile resistive switching behavior. However, the dynamic process of learning and memory is partially non-volatile, but also partially volatile, meaning that it decays with time to some extent, but not to its initial state. Therefore, it should be difficult to biorealistically mimic the Pavlovian conditioning with the detailed learning process with those non-volatile memristive devices, especially, in the aspect of learning frequency and recovery.

Here we investigated the hardware implementation of the Pavlovian conditioning based on the neuromorphic engineering with Ni/Nb-SrTiO_3_/Ti memristive devices whose resistive switching is partially non-volatile and whose conductance can be finely controlled. With the consideration of the natural forgetting phenomenon of the device in biological and physical senses, the three features of the Pavlovian conditioning, namely, acquisition, extinction and recovery, are implemented in the present device with the details of temporal contiguity, learning frequency and learning history. This work clearly demonstrates that partially non-volatile memristive devices with inherent dynamic properties can contribute to realizing higher-order complex neuromorphic functions.

## Results and Discussion

**Figure 1:**
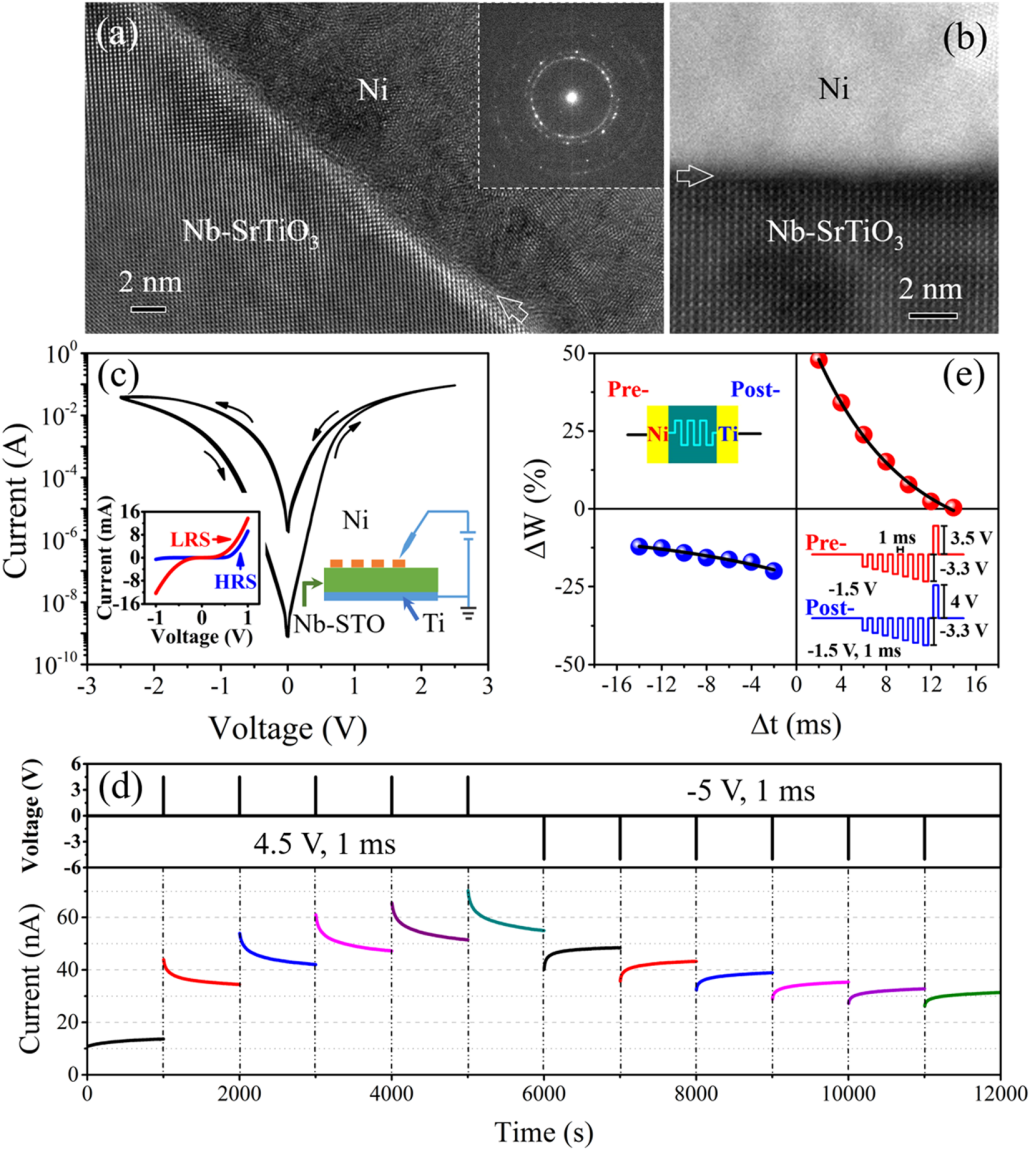
Device structure and electrical characterization. (**a**) HRTEM image of the interface, viewed along the [010] zone axis of Nb-SrTiO_3_. SAED of the Ni electrode in the inset shows the pattern of rings, demonstrating the polycrystalline feature of Ni. (**b**) Atomic-resolution HAADF investigations of the interface, viewed along the [010] zone axis of Nb-SrTiO_3_. (**c**) 50 cycles of current-voltage sweeping of the Ni/Nb-SrTiO_3_/Ti memristive device. The device structure is shown in the right inset. The device in HRS shows obviously rectifying characteristic while the one in LRS does not, as illustrated in the left inset. (**d**) Consecutive change of partially non-volatile conductance after pulse stimuli, exhibiting synaptic plasticity similarity. The read voltage is 0.05 V. (**e**) Change of the device conductance (Δ*W*) *vs*. the relative timing (Δ*t*) of pre- and post-spike, showing the STDP behavior. The conductance change can be considered as the change of the synaptic weight.

The present memristive device mainly consists of the Ni/Nb-SrTiO_3_ interface with Schottky barrier due to the high work function of Ni (5.15 eV) and the ohmic contact at the Ti/Nb-SrTiO_3_ interface, while Nb-SrTiO_3_ is conductive^40^. Structural properties of the Ni/Nb-SrTiO_3_ interface were investigated; Fig. 1a is the high-resolution transmission electron microscopy (HRTEM) image of the Ni/Nb-SrTiO_3_ interface, viewed along the [010] zone axis of Nb-SrTiO_3_. Selected area electron diffraction (SAED) of the Ni electrode shows the pattern of rings, as inserted in Fig. 1a, demonstrating that the Ni electrode exhibits a polycrystalline feature. In addition, atomic-resolution high-angle annular dark-field (HAADF) investigations of the Ni/SrTiO_3_ interface were performed, viewed along the [010] zone axis of Nb-SrTiO_3_ as well, the result is shown in Fig. 1b. The Ni/Nb-SrTiO_3_ interface, as indicated by arrows in Fig. 1a,b, is free of nickel oxide.

The Ni/Nb-SrTiO_3_/Ti memristive devices exhibit stable bipolar resistive switching characteristics. 50 cycles of current-voltage sweeping were conducted and are shown in Fig. 1c, and the right inset illustrates the device structure. The device in the high resistance state (HRS) exhibits obviously rectifying characteristic, while the one in the low resistance state (LRS) does not, as shown in the left inset, indicating the modulation of the Schottky barrier during the resistive switching process. Lots of works reported competing resistive switching mechanisms, such as trapping/detrapping of defect states^40–43^ as well as oxygen ion migration^44–46^. Recently, Baeumer *et al*.^44^ demonstrated that the resistive switching behavior in the metal/Nb-SrTiO_3_ based memristive devices can be accounted for by the oxygen ion migration. Namely, a positive voltage applied to the top electrode attracts oxygen ions from the metal/Nb-SrTiO_3_ interface and induces the oxygen excorporation, while a negative voltage incorporates oxygen ions at the metal/Nb-SrTiO_3_ interface, modifying the Schottky barrier height^44^. In the present device, with the application of positive pulses (4.5 V, 1 ms), the conductance increases consecutively, while with the application of negative pulses (−5 V, 1 ms), the conductance decreases successively, as shown in Fig. 1d. It can be seen that the conductance of the device is partially non-volatile, and it spontaneously decays over time after each stimulus application, which can be ascribed to the reoxidation of a previously oxygen-deficient region^44^. Here, the timescale of about 1000 s is just an instance, and some works reported that by inserting different oxides, for example, yttria-stabilized ZrO_2_ and Al_2_O_3_, between top electrode and SrTiO_3_, the decay property can be affected^44, 47^. The successive conductance increase is analogous to learning, while the spontaneous conductance decay is similar to forgetting. Also, such a successive conductance change under electrical stimuli is quite suitable for mimicking synaptic plasticity^2, 8–10, 12, 14, 16, 48^.

The time-dependent long-term potentiation (LTP) in biological synapses consists of two periods with different characteristics, namely a spontaneous fast decay and a slow one that finally becomes stable^9, 49, 50^, which is the basis for forgetting and memory^10, 49, 50^. The synapse-like spontaneous decay enables the present artificial synapse to remember its past dynamic history, which is the key point to biorealistically mimic the Pavlovian conditioning. Furthermore, STDP, which relies on relative spike timings of presynaptic and postsynaptic neurons and encodes the relative timing information, is reported to play an important role in the Pavlovian conditioning^51, 52^. The Pavlovian conditioning is even regarded as an emergent property of a spatially extended, spiking neural circuit with STDP^53^. Here STDP was also realized by engineering the pre-spike and post-spike pulses with the widely adopted overlapping spiking pulse protocol^9, 14, 15^, which builds a connection between the temporal relation Δ*t* and the pulse amplitude^9, 15, 26, 54, 55^, as shown in Fig. 1e. For example, when Δ*t* equals to 2 ms, the maximum voltage is 6.8 V. The modification of the synaptic weight (here the device conductance) increases/decreases with decreased/increased relative timing Δ*t* between the pre-spike and post-spike, and the experimental data can be well fitted by an exponential function, which is the typical STDP characteristic of synapses^8, 9, 13^. The demonstration of the Pavlovian conditioning is based on such an asymmetric STDP.

**Figure 2:**
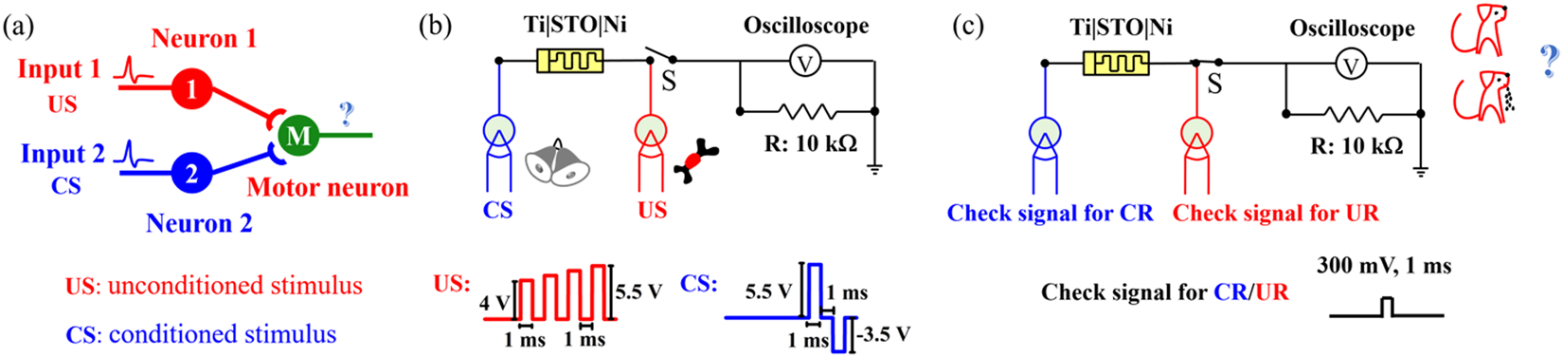
Pavlovian conditioning circuit design. (**a**) Prototype of the Pavlovian conditioning. After US reaches neuron 1, the neighbouring motor neuron responds to it; while in the case of CS, whether the motor neuron close to neuron 2 responds depends on the interconnection strength between them. (**b**) Memristive circuit with electrical US and CS to mimic the Pavlovian conditioning. CS is applied on the Ti electrode, while US is applied on the Ni electrode, and the oscilloscope is used to test the divided voltage of the resistor (10 kΩ), which reflects the response of the motor neuron. (**c**) Circuit with switch (*S*) in ON state to check whether CR/UR is elicited.

Figure 2a shows a prototype of the Pavlovian conditioning by a reductionistic strategy for biological systems and the synaptic strength change corresponds to the acquisition and extinction process^20, 26, 31, 32, 35^. The synapse between the neuron and the motor neuron plays a key role in the Pavlovian conditioning, whose strength determines whether the motor neuron responds. The synaptic strength between neuron 1 and the motor neuron is so strong that the neighbouring motor neuron always responds to US; while in the case of CS, whether the motor neuron responds depends on the synaptic strength between neuron 2 and the motor neuron. Initially, the synaptic strength between them is weak and the motor neuron does not respond to NS. After repeated training trials with NS right before US, the synaptic strength is potentiated and the motor neuron starts to respond when only NS is applied. Now NS can be termed as CS. Meanwhile, the potentiated synaptic strength can be depressed when only CS is applied.

A simple neuromorphic circuit, shown in Fig. 2b, was designed to demonstrate the Pavlovian conditioning. The memristor acts as a synapse and its two terminals accept NS/CS (“Bell”) and US (“Food”) signals, respectively. The divided voltage of the resistor (10 kΩ) is regarded as the response. In this circuit, the interconnection strength between neuron 1 and the motor neuron is very strong, because the terminal for US, i.e. the Ni electrode of the memristor, is directly connected to the resistor, while that between neuron 2 and the motor neuron is dependent on the resistance state of the memristive device, whose Ti electrode is the terminal for NS/CS. To check the synaptic strength change and the response *in-situ*, an oscilloscope, instead of a comparator, was used. How the simple neuromorphic circuit works is explained in the following.

Only when checking the response measured by the oscilloscope, the switch (*S*) is set to ON (Fig. 2c). Before training process, the memristive device is in HRS. A check signal of 300 mV is chosen; it is impossible for the check signal alone to tune the resistance of the memristive device. The divided voltage tested by the oscilloscope determines the response of the motor neuron. If the response signal is higher than 15% of the check signal, namely 45 mV, the UR or CR is defined as being elicited. To check UR, the check signal is applied on the Ni electrode of the memristor, the divided voltage of the resistor is then simply 300 mV, and then UR is elicited. To check CR, the check signal is applied on the Ti electrode of the memristor, the tested voltage is about 5 mV (not given) and CR is not elicited. For acquisition, paired CS and US are applied to potentiate the synaptic strength, while the switch (*S*) is set to OFF. Afterwards, the check signal is applied on the Ti electrode of the memristor to test whether CR is elicited with the switch (*S*) being ON. After successful acquisition, CS is applied alone on the Ti electrode of the memristor with the Ni electrode being grounded to lead the synaptic depression with the switch (*S*) in the OFF state, which results in the extinction. The recovery is realized by reacquisition after extinction. In this work, CS and US are designed with a sequence of pulses according to the overlapping spiking pulse protocol shown in the inset of Fig. 2b. The spike with a sequence of pulses of increasing magnitude, or other shaped pulse, such as triangular, is widely used in neuromorphic function implementations^9, 14, 15, 26, 54, 55^. When the memristor is in HRS, individual electric stimuli can only change the resistance of the device to a limited extent, because no distinctive threshold voltage exists for the resistive switching in such a memristor. Here, the application of paired CS and US is defined as training trials. The temporal relation (i.e., time gap between CS and US) and the time interval between individual training trials significantly affect the Pavlovian conditioning, which are discussed in the followings.

According to the Hebb’s law^19, 32, 39^ for the Pavlovian type of learning^20, 26^, the temporal contiguity of CS and US is a pre-requisite for achieving the Pavlovian conditioning. Moreover, the causality is emphasized that the spike applied on the pre-synapse must be prior to that on the post-synapse in the asymmetric STDP to potentiate the synaptic strength. Here CS acts as the pre-synaptic spike, while US as the post-synaptic spike in the reductionistic Pavlovian conditioning. The effect of the CS-US temporal relation on the strength of CR has been widely observed^36^. And it is the first feature of the acquisition that is to be demonstrated with this neuromorphic circuit.

**Figure 3:**
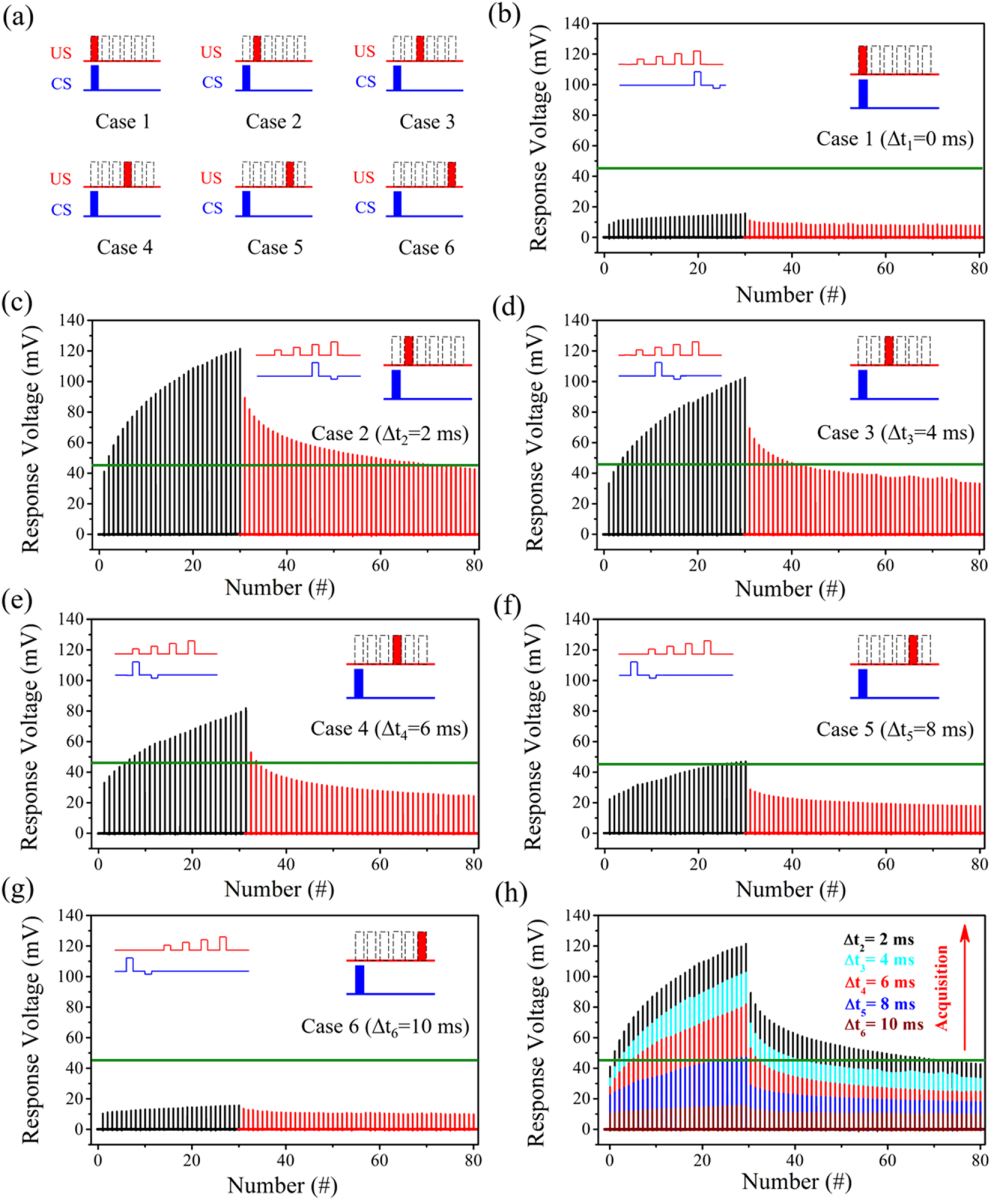
Experimental implementation of acquisition and extinction for the Pavlovian conditioning at different CS-US temporal relations. (**a**) Different cases of CS-US temporal relations. (**b–g**) Corresponding results of the Pavlovian conditioning experiments in different cases as indicated in the insets. The left inset is the applied training pulse pair, and the right inset shows the corresponding CS-US temporal relation. The black lines correspond to the acquisition and the red ones to the extinction. The number of training trials is 30, and the number of extinction trials is 50. The green line at 45 mV is the reference, above which the acquisition succeeds. (**h**) Comparison of all experimental cases in (**c–g**).

The cases of different CS-US temporal relations are exemplified in Fig. 3a. The CS and US are defined to be applied simultaneously in the case 1, namely the CS-US temporal relation Δ*t* equals to 0 s. Then, Δ*t* increases step by step for the cases of 2 to 6, where CS is always prior to US. In each case, the Ni/Nb-SrTiO_3_/Ti device was trained by 30 times of paired CS-US for acquisition, and subsequently underwent extinction trials by 50 times of CS alone. The 50 times of CS was just for a clear comparison. Normally, CS should be stopped when CR fails during extinction trials and then it becomes NS. The results of the Pavlovian conditioning experiments are given in Fig. 3b to g. The left inset is the training pulse pairs, and the right inset shows the corresponding Δ*t* in each figure. The black lines (the first 30 lines) indicate the response for the training process, while the red ones (the last 50 lines) are the response for the extinction process. The reference line of 45 mV, above which the acquisition succeeds, is indicated by the green line. It can be seen that when Δ*t* equals to 0 s, the acquisition is impossible, as shown in Fig. 3b, which is exactly due to the “predictive” nature of the Pavlovian conditioning^56^. When US precedes CS, the resistance of such a memristive device does not change much and the response voltage is small, which is designed on purpose by engineering US and CS. Only when CS is prior to US, the Pavlovian conditioning is possible. When CS slightly precedes US, namely Δ*t* equals to 2 ms, the speed of acquisition is high, and only 2 times of training trials are sufficient to achieve acquisition, as demonstrated in Fig. 3c. After 30 times of training trials, the strength of CR is so high that it takes many trials to extinguish CR. In the following cases of 3 to 6 where Δt equals to 4, 6, 8, 10 ms, respectively, similar acquisition and extinction processes are exhibited, as shown in Fig. 3d–g. It takes more training trials to elicit CR when Δ*t* becomes larger. It is also noted that CR cannot be elicited and the response voltage saturates with more training trials when Δ*t* is 10 ms. For a clear comparison, the results for the cases of 2 to 6 where CS precedes US are compiled in Fig. 3h. When CS and US are not in the proper Δ*t* window, CR cannot be elicited, because the CS and US voltages are designed to be not large enough.

**Figure 4:**
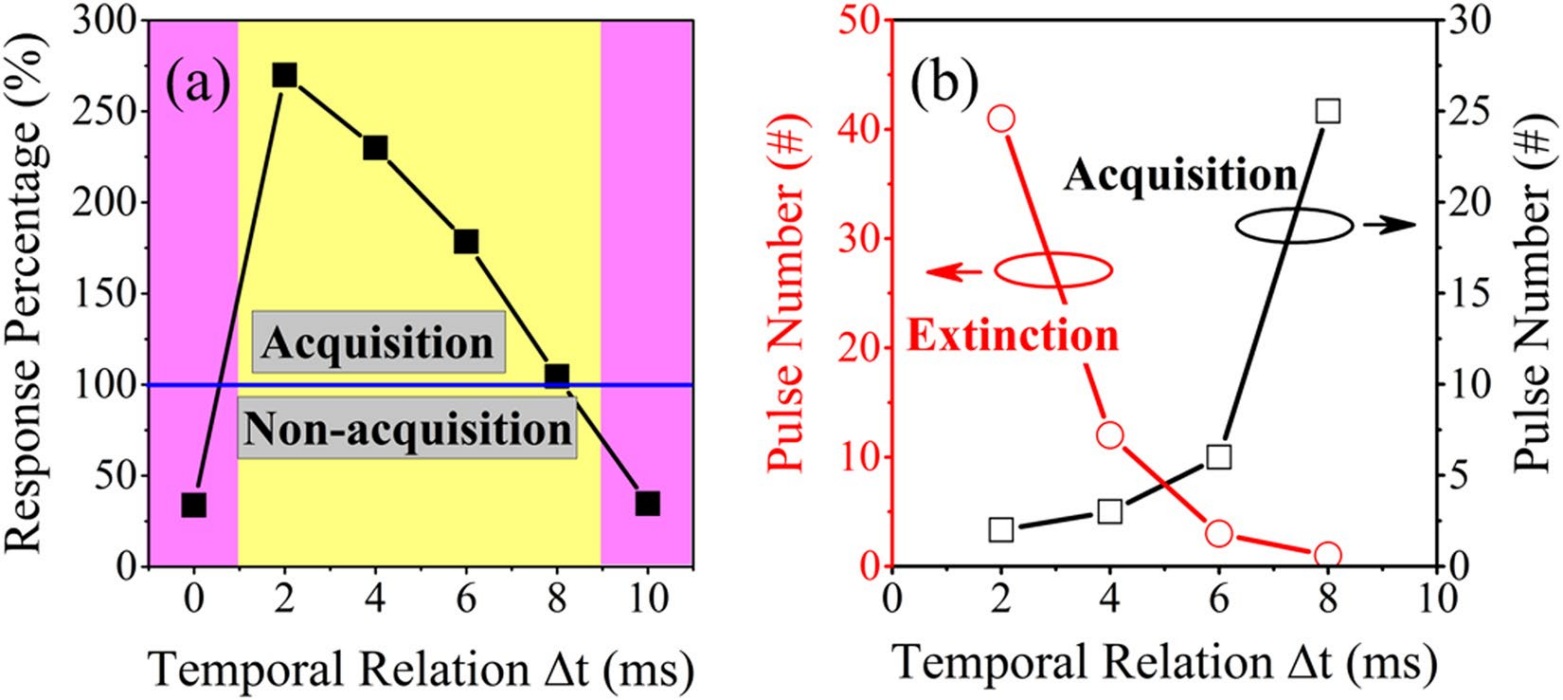
Effect of CS-US temporal relation on the Pavlovian conditioning. (**a**) The strength of CR (percentage of response compared to the reference) after 30 times of training trials at different temporal relations. (**b**) Number of training trials necessary for acquisition from the initial state and the number of extinction trials at different temporal relations.

Based on the above experiments, two important features related to the temporal relation can be extracted. According to Fig. 4a, for the fixed number of training trials, here 30 times, CR can be elicited only in a certain temporal relation range. In this range, for a shorter temporal relation, the resulted CR becomes stronger. However, when CS and US are applied simultaneously, or when US is far behind CS, CR can never be elicited. The required number of training trials for acquisition and the number of extinction trials, strongly depend on Δ*t*, as shown in Fig. 4b. In the Δ*t* window where acquisition can happen, the number of training trials necessary for acquisition increases with increasing temporal relation, while the number of extinction trials decreases.

**Figure 5:**
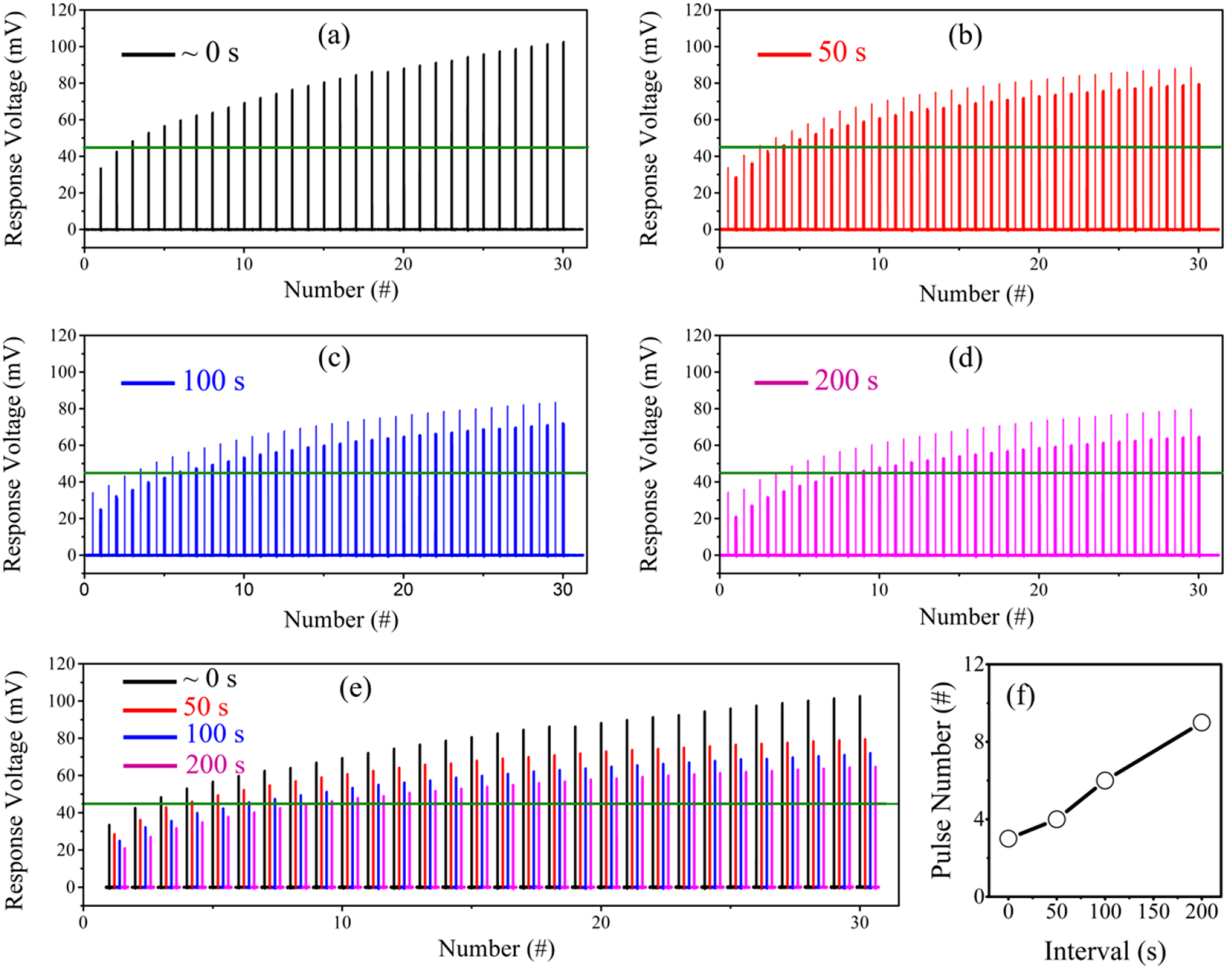
Effect of time interval between individual training trials on acquisition. The time intervals are approximately 0 s, 50 s, 100 s and 200 s in (**a**) to (**d**), respectively. The CS-US temporal relation is fixed to 4 ms. The coarse lines are the response voltage tested after the corresponding time interval. The fine lines are the voltage checked immediately after the stimuli. (**e**) Comparison of the response voltage with different time intervals in (**a**) to (**d**). Only coarse lines are compiled. (**f**) Necessary number of training trials for acquisition with different time intervals.

In the experiments presented in Figs 3 and 4, a CS-US pair was applied immediately after the previous one, i.e., the time interval between individual training trials is approximately 0 s. However, not only does the CS-US temporal relation matter to the Pavlovian conditioning, but also the interval between each training trial does^36^. It is known that the natural forgetting plays an important role in memory and learning. With the partially non-volatile property of the Ni/Nb-SrTiO_3_/Ti memristive device, the effect of the time interval between individual training trials on the Pavlovian conditioning is illustrated in Fig. 5. In this experiment, four time intervals, i.e., approximately 0 s, 50 s, 100 s, and 200 s, were chosen to demonstrate this issue, while the CS-US temporal relation Δ*t* was fixed to 4 ms. In each case, the response voltage was measured after the corresponding time interval, as shown by the coarse lines in Fig. 5a to d. In order to demonstrate the natural forgetting process, the response voltage was also checked immediately after applying the CS-US pairs; the voltages are shown by the fine lines in corresponding figures. The voltage difference between corresponding coarse and fine lines reflects the forgetting process. For comparison, the response voltages measured after different time intervals are compiled in Fig. 5e. It can be clearly seen that the response voltage decreases with increasing time interval. In other words, the more frequently Pavlov trained, the better his dog learned, which is exactly the very basic feature of learning. In addition, the number of training trials necessary for acquisition is also extracted, as illustrated in Fig. 5f, which increases with increasing time interval.

**Figure 6:**
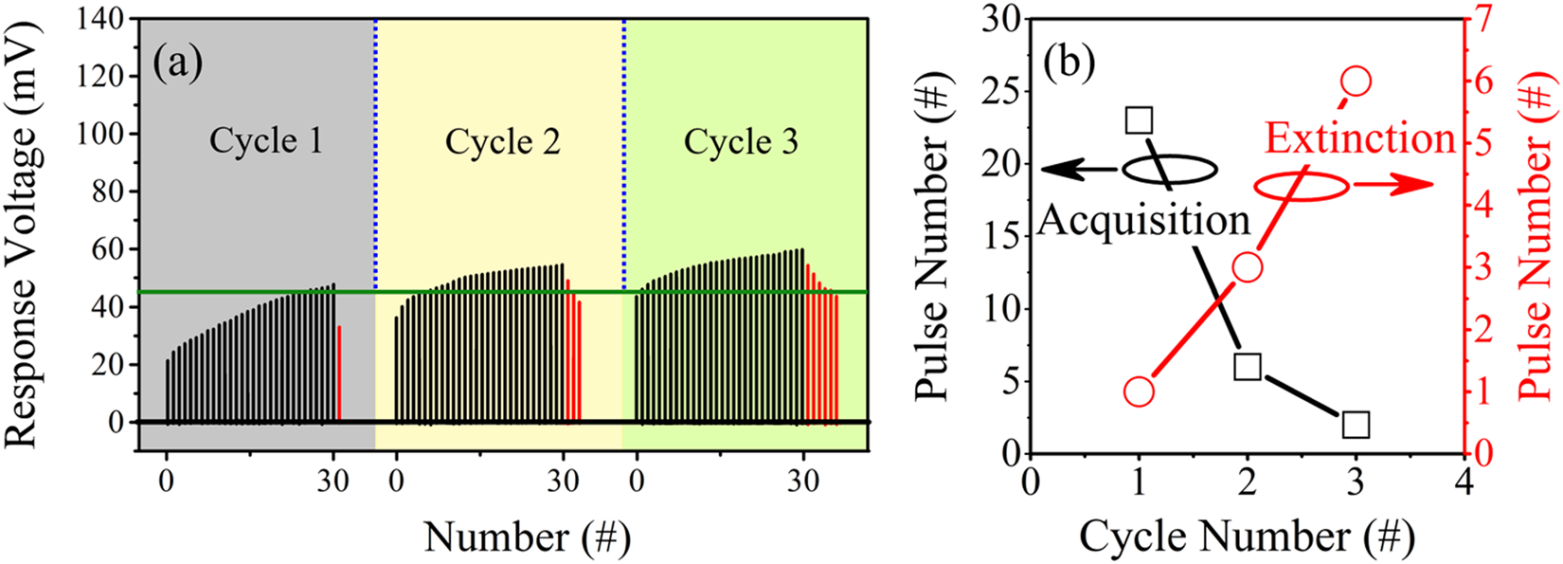
Recovery behavior of the Pavlovian conditioning. (**a**) The three consecutive cycles contain 30 times of training trials and certain times of extinction trials until CR fails, the CS-US temporal relation is 8 ms and the time interval between individual training trials is approximately 0 s. The number of training trials for acquisition decreases, while the number of extinction trials increases in the three consecutive cycles. (**b**) Comparison of the number of training trials for acquisition and the number of extinction trials in each cycle.

The next part is about the recovery behavior of the Pavlovian conditioning. In biology, the extinction procedure does not completely eliminate the effect of the conditioning. Therefore, reacquisition usually happens much faster than the previous one^38^, which is termed as recovery. In this experiment, the CS-US temporal relation was set to be 8 ms and the time interval between each training trial approximately 0 s. Three consecutive acquisition and extinction cycles were performed. Each acquisition contained 30 times of training trials, while each extinction contained a certain number of extinction trials until CR failed, at this point CS became NS. The results are given in Fig. 6a. The strength of response after 30 times of training trials increases in the three consecutive cycles, and the number of training trials necessary for acquisition decreases, while the number of extinction trials increases as shown in Fig. 6b, which shows a typical recovery behavior of the Pavlovian conditioning.

Here, the time interval between pulses of US and CS and the temporal relation Δ*t* are on the scale of *ms*; if the values are proportionally increased to the scale of *s*, the three basic features of the Pavlovian conditioning (acquisition, extinction and recovery) would not be changed.

Usually the features of the Pavlovian conditioning can be interpreted based on the synaptic plasticity^31, 39^. In biological systems, the synaptic plasticity is modulated by spikes and controlled by the activity of neurons (i.e., frequency and relative timing of the spikes), which is due to, for example, the postsynaptic calcium ion (Ca^2+^) concentration^13, 57^. The spike increases the Ca^2+^ concentration, afterwards the natural decay of the Ca^2+^ concentration follows, which provides an internal timing mechanism to encode the activity information on the spikes. Also, the efficacy of the synaptic transmission increases upon potential spikes, but spontaneously decreases subsequently. Initially, the synaptic strength between neuron 2 and the motor neuron (Fig. 2a) is weak, and NS cannot trigger the motor neuron. After repeated training trials with the CS-US pairs, the synaptic strength increases, so that CR occurs. Meanwhile, the time interval between training trials, or frequency of training trials, also affects the synaptic strength, consequently, the Pavlovian conditioning.

Initially the SrTiO_3_ based memristive device is in HRS, for acquisition, the memristive device should change to LRS, while for extinction, the device should return to HRS. Upon applying positive pulses, the conductance of the device increases, but the conductance decays subsequently (Fig. 1d); therefore, the memristive device is partially non-volatile. Even though it is different from a biological synapse in mechanism, the partial non-volatility of the memristive device is suitable for mimicking the synaptic plasticity. In the experimental implementation of the Pavlovian conditioning, a CS-US pair can be regarded as overlapped spiking pulses, and in fact, result in effective positive voltage pulses to the Ni electrode. Therefore, the CS-US pair changes the resistance state of the device to LRS and the acquisition is achieved after repeated training trials. The CS alone can be considered as an effective negative voltage pulse applied on the Ni electrode. When the resistance state of the device is in LRS, the voltage pulse of CS can change the resistance state of the device to HRS and results in extinction. However, when the device has a comparatively high resistance, the CS alone cannot increase the resistance remarkably. The resistance change of a memristor is not only dependent on the stimulus, but also on the initial resistance state of the memristor^58^.

As for acquisition, different CS-US temporal relations result in overlapped spiking pulses with different voltage amplitudes, thus, different effects on acquisition, as demonstrated in Fig. 3. With increasing time interval between individual training trials, the device conductance decays to lower levels, resulting in smaller conductance before subsequent training trial. Therefore, increasing the time interval between individual training trials delays the acquisition, as illustrated in Fig. 5. Since the conductance cannot completely decrease to the initial state after extinction, the recovery becomes faster, as shown in Fig. 6.

It should be mentioned that it is hard to realize another important feature of the Pavlovian conditioning, i.e., blocking^59^. Blocking is that a conditioned stimulus (CS1) and US is impaired if the CS1 is presented together with a second CS (CS2) that has already been associated with the US during the conditioning process. In this circuit, as long as the association between CS and US is built, the memristive device is in the low resistance, which makes the blocking impossible. The Ni/Nb-SrTiO_3_/Ti memristive device with intrinsic forgetting properties is quite suitable for mimicking the neuromorphic function^9, 10, 12, 13^, and the realization of the blocking may be possible by improving the simple circuit.

In summary, with the Pavlovian conditioning, a biological body is able to learn and remember the relationship between unrelated information, which is an important characteristic of a biological brain. The Pavlovian conditioning with three basic features, namely acquisition, extinction and recovery, is mimicked with memristive devices based on SrTiO_3_. For acquisition, CS should precede US but not be too far prior to US, a certain CS and US temporal relation range is required. Within this range, a shorter temporal relation results in a faster acquisition. The increasing time interval between individual training trials results in less strength of response and more number of training trials for acquisition, which is due to the intrinsic decay nature of the Ni/Nb-SrTiO_3_/Ti memristive device. For extinction, repeated CS alone can depress the device conductance and make CR fail in the end. Since the device is unable to thoroughly go back to the initial state within a limited time period after extinction, the reacquisition or recovery is faster than the previous one. With the successful implementation of the major features of the Pavlovian conditioning in the memristive device, this work shows a straightforward example for the neuromorphic application of memristive devices.

Although the Pavlovian conditioning was mimicked with SrTiO_3_ based memristive devices, the significance of this work is not restricted to SrTiO_3_. The key feature of the memristive device is that its resistive switching is partially non-volatile, which is phenomenologically similar to the forgetting process in a biological brain. This work demonstrates that partially non-volatile memristive devices with inherent forgetting properties are very promising for imitating the brain’s learning paradigm.

## Methods

### Device fabrication

(100)-oriented SrTiO_3_ single crystals (5 × 5 × 0.5 mm^3^) doped with 0.1 wt% Nb (CrysTec, Germany) were used. The top Ni electrode and the bottom Ti electrode were prepared by sputtering in Ar.

### Structural characterization

Cross-sectional specimens for structural investigations were prepared by focused ion beam method (FEI Helios600i FIB/SEM). Transmission electron microscopy (TEM) and high-angle annular dark field (HAADF) investigations on the Ni/SrTiO_3_ interface were carried out on a JEM ARM200F spherical-aberration corrected electron microscope, operated at 200 keV.

### Device and functional characterization

All the measurements were conducted with a Keithley 4200 Semiconductor Characterization System connected with a Cascade SUMMIT 11000B semi-automatic probe station in air and at room temperature.

When the check signal of 300 mV for CR is applied on the Ti electrode, the voltage response of the motor neuron represented by the divided voltage *V*_R_ across the resistor (10 kΩ), can be obtained according to the following equation:

1 $${V}_{R}={V}_{C}\times \frac{R}{R+{R}_{M}}$$

Here *V*_*C*_ is the check signal of 300 mV, *R* is the resistance value of the resistor, and *R*_*M*_ is the resistance of the memristor. When *V*_*R*_ is larger than 45 mV, CR is defined as being elicited. *R*_*M*_ decreases during the training process for acquisition, and increases during the extinction process. Owing to the non-linearity of the memristor, the resistance of the memristor varies under different voltages. But only when *R*_*M*_ decreases to a certain extent, the voltage across the resistor becomes larger than 45 mV. When the check signal of 300 mV for UR is applied on the Ni electrode, the voltage across the resistor is just 300 mV. Thus, UR is elicited directly.
